# Evaluation of the Systemic Inflammation in Patients with Bell’s Palsy: Monocyte-to-High-Density Lipoprotein Cholesterol Ratio and Hematologic Indices of Inflammation

**DOI:** 10.3390/jcm14176194

**Published:** 2025-09-02

**Authors:** Demet Aygun, Mustafa Ibas, Hafize Uzun

**Affiliations:** 1 Department of Neurology, Faculty of Medicine, Istanbul Atlas University, 34408 Istanbul, Turkey; 2 Department of Otorhinolaryngology-Head and Neck Surgery, Faculty of Medicine, Istanbul Atlas University, 34408 Istanbul, Turkey; ibasmustafa@gmail.com; 3 Department of Medical Biochemistry, Faculty of Medicine, Istanbul Atlas University, 34408 Istanbul, Turkey; huzun59@hotmail.com

**Keywords:** Bell paralysis, facial nerve paralysis, prognosis, neuroinflammation, monocyte-to-high-density lipoprotein cholesterol ratio

## Abstract

**Background**: We aimed to investigate the prognostic significance of hematological inflammatory indices and monocyte-to-high-density lipoprotein cholesterol ratio (MHR) in the diagnosis and prognosis of Bell’s palsy. **Method**: The study included 156 cases diagnosed with Bell’s palsy in the neurology clinic and 156 healthy controls. The patients diagnosed with Bell’s palsy were staged according to the House–Brackmann Scoring system. Hematological inflammatory parameters such as neutrophil-to-lymphocyte ratio (NLR), platelet-to-lymphocyte ratio (PLR), systemic immune-inflammation index (SII) and systemic inflammation response index (SIRI) levels, and MHR were calculated from the parameters in the patient files. **Result**: Hematological inflammatory parameters such as NLR, PLR, SII, SIRI, and MHR were found to be higher in Bell’s palsy patients. In addition, these parameters were found to be higher in patients with grade V and above Bell’s palsy and in patients who did not respond to treatment, compared to the grade IV group and patients who responded to treatment, respectively. SIRI was an independent predictor of both the diagnosis of the disease and the lack of response to treatment, and this was confirmed by LASSO analysis. **Conclusions**: This study is among the few that demonstrated predictive models based on hematological inflammatory indices that can aid in both the diagnosis and treatment response assessment of newly diagnosed Bell’s palsy patients, validated using the LOOCV method. The findings highlight the potential clinical utility of simple, inexpensive, and practical biomarkers such as NLR, PLR, SII, SIRI, and MHR. These easily accessible parameters may support early diagnosis and prognostic evaluation in routine clinical settings.

## 1. Introduction

Bell’s palsy is the most common cause of facial nerve paralysis. It is an idiopathic peripheral facial paralysis characterized by acute unilateral weakness of the facial muscles [1,2]. In epidemiological studies, the frequency of Bell’s palsy is thought to be between 11 and 100,000 [3]. It can be seen in all age groups, mostly heals completely, and can recur in 15% of cases with different severities. Clinical findings include paralysis of all muscles in the affected facial area, impaired taste sensation, pain radiating to the postauricular region, and numbness in the face, which may accompany other symptoms [4]. Although these findings are sufficient for diagnosis, hemogram, biochemistry, and central nervous system and ear-related pathologies must be excluded before treatment to exclude secondary causes [5].

The etiology of Bell’s palsy, although there is no clearly defined risk factor, genetic, immunological, vascular, and infectious etiologies are the most common suggested causes [6,7,8]. Recently, some autoimmune processes and viral infections have also been blamed [9,10]. In case-based evaluations, the herpes simplex type 1 virus is associated with recent periods [11,12,13,14]. In pathogenesis, as a result of the inflammatory process, inflammation of the facial nerve sheath and edema of the fallopian tubes, especially in the labyrinth segment, occur [15,16].

Neutrophils, the main players in systemic inflammation, produce neutrophil elastases, matrix metalloproteinases, and various cytokines. Lymphocytes have anti-inflammatory properties. Neutrophil-to-lymphocyte ratio (NLR), platelet-to-lymphocyte ratio (PLR), systemic immune-inflammation index (SII), and systemic inflammation response index (SIRI) have been used as markers of systemic inflammation in recent years. Monocytes are the main source of prooxidant and proinflammatory cytokines. HDL-cholesterol exerts anti-inflammatory and antioxidant effects. The combination of these parameters, monocyte/HDL cholesterol ratio (MHR), neutrophil/HDL cholesterol ratio (NHR), and lymphocyte/HDL cholesterol ratio (LHR) are novel biomarker associated with inflammation [17,18,19,20,21,22,23,24].

Inflammation plays a central role in Bell’s palsy by causing immune-mediated facial nerve injury. Neutrophils promote inflammation through proteolytic enzymes and cytokines, while lymphocytes have anti-inflammatory effects; thus, the NLR reflects this balance. Monocytes contribute to oxidative stress, whereas HDL cholesterol provides antioxidant protection, making the MHR a useful systemic inflammation marker. Composite indices such as PLR, SII, and SIRI capture complex immune interactions and may have prognostic significance in Bell’s palsy [17,18,19,20,21,22,23,24].

Therefore, we hypothesized that alterations in hematological inflammatory parameters such as NLR, PLR, SII, SIRI, and MHR occur in Bell’s palsy, and that heightened inflammation plays a role in its etiopathogenesis. This study aims to evaluate the role of systemic inflammation in the pathogenesis of Bell’s palsy by analyzing hematological inflammatory markers, including NLR, PLR, SII, SIRI, and MHR, and to investigate their potential prognostic value in diagnosis and clinical assessment.

## 2. Methods

This study was planned as a retrospective case-control study at the Atlas University Faculty of Medicine, Neurology Clinic. The study was designed by the Good Clinical Practices protocol and the Declaration of Helsinki. Ethics committee approval was obtained from the Istanbul Atlas University non-invasive scientific research ethics committee (Ethics Committee approval number: E-22686390-050.01.04-6988, Date: 10 August 2021).

### 2.1. Study Design and Population

Cases who applied to the neurology outpatient clinic with facial paralysis between January 2020 and January 2021 or were diagnosed with Bell’s palsy after consultation, who did not have peripheral facial paralysis before, who applied within the first seven days of their complaints, and who were between the ages of 12 and 60 were included in the study, respectively. The control group was composed of healthy individuals who met the inclusion criteria among the cases examined in the check-up program in the hospital database and were compatible with the age and gender of the patients included in the study. Patients with a chronic disease such as hypertension, diabetes mellitus, chronic renal failure, chronic liver failure, autoimmune disease, hematological disease, malignancy, pregnancy, heart failure, those with findings other than peripheral facial paralysis in neurological, ear-nose-throat and systemic examinations, those with a history of surgical intervention within the last month, those with an infection within the last two weeks, those with intracranial facial nerve pathology detected in cranial imaging, those using steroids, antibiotics, antivirals, antiaggregant, anticoagulants and immunosuppressants, and those who had started treatment were excluded from the study. After applying the exclusion criteria, 156 patients and 156 age- and gender-matched controls were included in the analysis.

To ensure homogeneity of the study groups and to exclude inflammatory, infectious, or malignant conditions, all participants underwent a detailed review of their medical history and clinical examination. Chronic diseases such as hypertension, diabetes mellitus, chronic renal or liver failure, autoimmune diseases, hematological disorders, and malignancies were identified and excluded based on patient records and history. Additionally, individuals with clinical signs or symptoms of acute infection (including fever or recent infectious episodes within the last two weeks), laboratory abnormalities suggestive of infection (such as elevated white blood cell count), or use of medications affecting inflammatory status (including corticosteroids, antibiotics, antivirals, antiaggregants, anticoagulants, and immunosuppressants) were excluded. This comprehensive screening was performed retrospectively using electronic medical records and laboratory data to minimize confounding factors affecting systemic inflammation markers.

Clinical and demographic data, including prior treatment history, were retrospectively obtained from electronic medical records and confirmed through patient interviews to ensure accuracy.

### 2.2. Clinical Evaluation of Facial Nerve Functions

Clinical evaluation of Bell palsy side involvement and facial nerve functions was recorded from the files by staging according to the House–Brackmann Scoring (HBS) system, which was started to be used as a standard by the American Academy of Otolaryngology Facial Nerve Diseases Committee [25]. The HBS system classifies facial nerve function into six stages: Stage 1 (Normal), Stage 2 (Mild loss of function), Stage 3 (Moderate loss of function without deformity), Stage 4 (Moderate loss of function with deformity), Stage 5 (Severe loss of function), and Stage 6 (Complete paralysis). Upon their initial presentation to our hospital, patients exhibited facial motor function classified as H-B grade IV to VI.

All patients were treated with corticosteroids (1 mg/kg/day prednisone as the initial dose), with a gradual dose decrease continued for at least two weeks. After treatment, patients who achieved H-B grade II were considered to have responded successfully to corticosteroid therapy (recovery group), whereas those with H-B grade IV to VI were classified as the non-recovery group.

### 2.3. Hematological Inflammatory Parameters

In the neurology clinic, patients diagnosed with Bell’s palsy are routinely evaluated for hematological and biochemical parameters by taking a blood sample from the antecubital vein at around 8 a.m. after fasting for at least 8 h. Hematological parameters are analyzed in the same laboratory using the CELL-DYN 3700 SL (Abbott Diagnostics, Chicago, IL, USA) device. NLR is obtained by the ratio of the neutrophil level to the lymphocyte level, PLR is obtained by the ratio of the platelet level to the lymphocyte level, and ELR is obtained by the ratio of the eosinophil level to the lymphocyte level. SII was obtained by multiplying the platelet count by the neutrophil count and dividing by the lymphocyte count (platelet × neutrophil/lymphocyte). SIRI was obtained by multiplying the monocyte count by the neutrophil count and dividing by the lymphocyte count (monocyte × neutrophil/lymphocyte). The MHR is calculated as the ratio of monocyte count (10^3^ cells/L) to HDL cholesterol (mg/dL). 

### 2.4. Statistical Analysis

Normality was assessed using the Kolmogorov–Smirnov test. Normally distributed numerical variables are presented as mean ± SD, and non-normally distributed variables as median [IQR: Q1–Q3]. Categorical variables are reported as counts and percentages. Student’s *t*-test and Mann–Whitney U test were used for normally and non-normally distributed numerical variables, respectively. Chi-square, Yates’ correction, or Fisher’s exact test was applied for categorical data. ROC curve analyses were performed for each biomarker for different outcomes, including diagnosis, disease severity, and treatment response. LASSO regression identified key inflammatory markers associated with Bell’s palsy and non-recovery, with model performance evaluated using LOOCV; hyperparameter tuning was performed within each resampling loop, and repeated 10-fold cross-validation was conducted as a sensitivity analysis. In addition to classical indices (NLR, PLR, SII, SIRI), MHR was evaluated. Variable importance in projection (VIP) scores > 1.3 were considered effective in constructing latent factors. Significance was set at *p* < 0.05.

## 3. Results

The mean age of Bell’s palsy patients was 46.3 ± 13.2 years, with males comprising 66.7% of the cohort. The left side was predominantly affected, and half of the patients were classified as H-B stage V. While the demographic characteristics of the patient and control groups were similar, their hematological and metabolic parameters showed notable differences.

In comparison to the control group, Bell’s palsy patients exhibited significantly higher median values for CRP (4.0 vs. 1.4 mg/dL, *p* < 0.001), PLR (115.7 vs. 90.6, *p* = 0.008), NLR (2.0 vs. 1.2, *p* < 0.001), SII (452.2 vs. 233.4, *p* < 0.001), and SIRI (1.2 vs. 0.5, *p* < 0.001), as well as MHR (0.01 vs. 0.01, *p* < 0.001) (Table 1).

**Figure 1:**
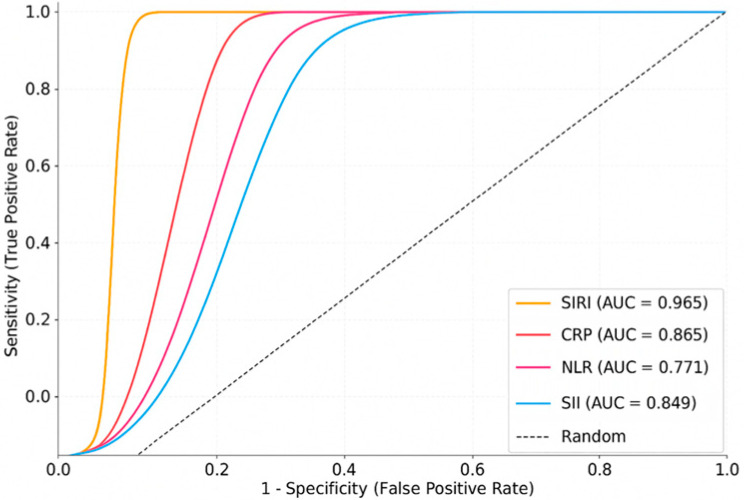
ROC curves for differentiating Bell’s palsy vs. healthy controls.

**ROC analysis** performed to assess the diagnostic accuracy of individual markers showed that SIRI had the highest discriminatory power (AUC: 0.965; 95% CI: 0.947–0.983), followed by CRP (AUC: 0.865), SII (AUC: 0.849), and NLR (AUC: 0.771). LASSO regression similarly identified SIRI and CRP as the most influential variables in the multivariable model, underscoring their diagnostic relevance (Figure 1, Table 2). These ROC analyses were performed using individual biomarkers in a univariable manner.

A LASSO regression analysis was performed to identify the most relevant inflammatory markers associated with the outcome. The model demonstrated a strong predictive capacity, with 89% of the variation explained by the predictor variables (inflammatory mediators) and 70% of the variation explained for the outcome variable. The model used a single latent factor and achieved a high AUC of 0.98, indicating excellent discriminatory ability. Furthermore, the model correctly classified 92.1% of cases, with a *p*-value < 0.001, confirming statistical significance. Among the inflammatory markers, SIRI (VIP: 3.97), CRP (VIP: 2.50), and MHR (VIP: 1.41) were found to have the strongest positive associations with the outcome.

Table 3 compares the performance of the LASSO and LOOCV methods for predicting Bell’s palsy using a multivariable regression model. With LOOCV, the area under the curve (AUC) was 0.97 (standard error [SE] 0.02; 95% confidence interval [CI] 0.93–1.00). The sensitivity, specificity, positive predictive value (PPV), and negative predictive value (NPV) were 84.0%, 97.4%, 97.0%, and 85.9%, respectively, leading to an overall correct classification rate of 90.7%. In contrast, the LASSO method showed an AUC of 0.96 (SE 0.02; 95% CI 0.93–1.00) with a sensitivity of 84.6%, specificity of 97.4%, PPV of 97.2%, and NPV of 86.4%. The LASSO approach correctly classified 92.1% of cases.

**Figure 2:**
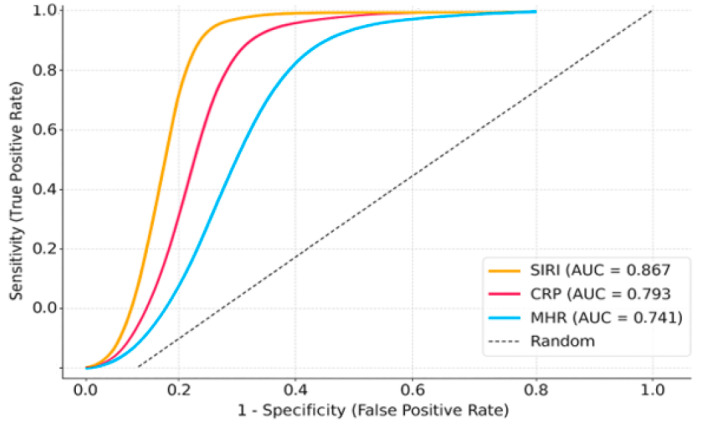
ROC curves for predicting treatment response (recovered vs. non-recovered).

In comparison to the stage IV group, stage V–VI patients exhibited higher inflammatory indices, including CRP, NLR, SII, SIRI, and MHR (Table 4). For the comparison of disease severity (Stage V–VI vs. Stage IV), ROC analyses were conducted separately for each biomarker. ROC analysis to differentiate severe disease (Stage V–VI vs. IV) demonstrated that SIRI had the strongest prognostic power (AUC: 0.867), followed by NLR (AUC: 0.813), CRP (AUC: 0.793), and MHR (AUC: 0.741), indicating these markers can effectively predict initial disease severity (Figure 2).

Comparison of demographic and laboratory findings by the House–Brackmann Scoring system in patients with Bell’s palsy is presented in Table 5.

Diagnostic performance and ROC curves for predicting treatment response are presented in Table 6 and Figure 2.

There were no statistically significant differences in gender distribution or age between recovery and non-recovery groups. Red blood cell indices, including hemoglobin, RBC count, hematocrit, MCV, MCH, and MCHC, were also similar between groups (*p* > 0.05 for each parameter). The non-recovery group had higher total leukocyte counts, neutrophil counts, monocyte counts, plateletcrit, and platelet distribution width (PDW), as well as elevated PLR, NLR, SII, SIRI, and MHR (*p* < 0.05 for each parameter). Meanwhile, their lymphocyte counts were lower (*p* = 0.001) (Table 7).

**Figure 3:**
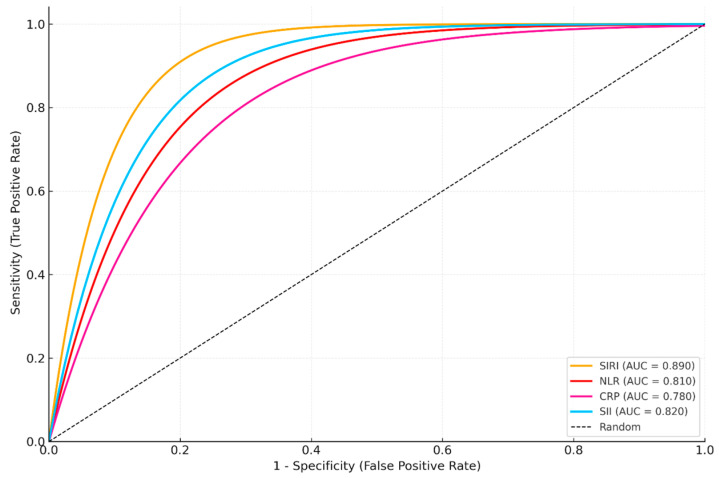
ROC analysis for treatment response prediction (recovery vs. non-recovery) indicated that SIRI had the highest prognostic accuracy, followed by SII.

For the treatment response outcome (recovery vs. non-recovery), ROC analyses were again conducted in an univariable manner for each biomarker. ROC analysis for treatment response prediction (recovery vs. non-recovery) indicated that SIRI had the highest prognostic accuracy (AUC: 0.890), followed by SII (AUC: 0.820) (Figure 3, Table 8). LASSO regression for the same outcome also selected SIRI and SII as the most influential markers, confirming their prognostic relevance.

The LASSO regression model for the same outcome evaluated the relative importance of all biomarkers simultaneously. Table 9 shows the LASSO analysis identifying key inflammatory markers related to non-recovery in patients with Bell’s palsy. A single latent factor accounted for 97% of the variance in the predictor variables (inflammatory mediators) and 50% of the variance in the outcome measures. The final model exhibited a high discriminative performance, with an AUC of 0.86 and correctly classified 93.8% of cases (*p* < 0.001). After adjusting for age and gender, the most influential markers associated with the recovery outcome were the SIRI (VIP = 1.65), CRP (VIP = 1.44), all showing positive or inverse associations with recovery. These findings underscore the importance of systemic inflammation in predicting non-recovery among patients with Bell’s palsy.

## 4. Discussion

This study is one of the rare studies showing that models that predict both the diagnosis of the disease and the response to treatment in newly diagnosed Bell’s palsy patients with hematological inflammatory parameters were validated by the LOOCV method. Hematological inflammatory parameters such as NLR, PLR, SII, SIRI, and MHR were found to be higher in Bell’s palsy patients. In addition, these parameters were found to be higher in patients with stage V–IV and above Bell’s palsy and in patients who did not respond to treatment, compared to the stage IV group and patients who responded to treatment, respectively. SIRI was an independent predictor of both the diagnosis of the disease and the lack of response to treatment, and this was confirmed by LOOCV. The inflammatory biomarkers evaluated in this study, including NLR, PLR, SII, SIRI, and MHR, are accessible and cost-effective parameters derived from routine blood tests. Their association with Bell’s palsy suggests potential utility as diagnostic and prognostic tools. Clinically, these markers could help identify patients with heightened systemic inflammation who may be at risk for poorer recovery, guiding treatment intensity and follow-up strategies. Moreover, monitoring these indices over time may provide insights into therapeutic response, offering a non-invasive method to tailor patient management. However, prospective studies are needed to validate their clinical applicability.

An increasing number of studies are examining the prognostic significance of hematological inflammation indices, which serve as indicators of this inflammatory process, in the diagnosis and treatment of Bell’s palsy [10,20,21,22,23,24,26,27,28,29,30,31,32,33]. For instance, Kim et al. [23] showed that higher NLR correlates with more severe facial paralysis and delayed recovery, paralleling our finding that patients with higher House–Brackmann stages had increased inflammatory indices [25]. However, some studies report inconsistent associations, likely due to smaller cohorts and varying disease severities [29,30]. Our relatively large sample size (*n* = 156) and standardized staging strengthen the validity of our conclusions. In a study conducted by Ulusoy et al. [28] on 24 patients with Bell’s palsy, the NLR rate was found to be higher in the Bell’s palsy group compared to the healthy control group. In the same study, no significant correlation was found between the NLR level and the prognosis of Bell’s palsy. In patients who completely recovered during follow-up in Bell’s palsy, platelet levels were found to be lower compared to patients who did not recover. The platelet distribution width (PDW), one of the platelet indices, was found to be higher in patients who completely recovered after Bell’s palsy compared to patients who did not recover [28].

In a study conducted by Sahin et al. [29] with 28 Bell’s palsy patients, NLR levels were found to be higher in the patient group compared to the control group, and no significant difference was found between the groups in terms of PLR and MPV levels. No significant difference was found in the patient group in terms of hematological parameters at presentation and in the first week of follow-up. In the same study, no significant difference was found in terms of hematological parameters in the stage 2 and 3 Bell’s palsy groups [32]. In a study conducted by İnan et al. on 79 Bell’s palsy patients, the NLR level was found to be higher in the patient group compared to the control group. It was determined that the NLR level in the Bell’s palsy group had prognostic importance for complete recovery of the disease [29]. In a study conducted by Atan et al. [20] on 99 patients with Bell’s palsy, NLR and PLR levels were found to be higher in the patient group compared to the control group. No significant relationship was found between the stage of Bell’s palsy and NLR and PLR levels [34]. In a study conducted by Kim et al. [23] on 51 Bell’s palsy patients, NLR levels were found to be higher in patients with severe Bell’s palsy compared to those with moderate and mild Bell’s palsy. The time for complete recovery of the disease was found to be longer in the group with high NLR levels compared to the group with low NLR levels [23]. In a meta-analysis conducted by Oya et al. [30], NLR levels were found to be higher in the Bell palsy group compared to the healthy control group. NLR levels were found to be lower in patients who recovered completely compared to those who did not. High NLR levels were found to be associated with poor prognosis and severity of facial nerve inflammation. However, no significant difference was found between the groups in terms of PLR levels in the same study [30].

In our study, NLR and PLR levels were found to be higher in the Bell’s palsy group compared to the healthy control group. When we examine the above studies, we see that in some of them, these parameters were like our study in the patient and healthy control groups; in some, they were found to be completely different. In some studies, NLR levels may have been high in the patient group while PLR levels were normal or vice versa. Several factors may contribute to this condition. In many studies, the number of patients included in the study was limited, and very few patients were included. In contrast to these studies, 156 patients with Bell’s palsy were included in our study. Another reason is that there are serious differences in the H-B stages of the patients included in the studies. We know that inflammatory parameters are higher in patients with a high H-B stage in many studies. This may cause the results to be different. The results of our study also support this claim. Because NLR and PLR levels were found to be higher in those with a disease stage above V–VI compared to stage IV. Our study confirms that hematological inflammatory markers—NLR, PLR, SII, SIRI, and MHR are significantly elevated in patients with Bell’s palsy compared to healthy controls, and correlate with both disease severity and treatment response. Notably, SIRI emerged as an independent predictor for diagnosis and lack of treatment response, findings robustly validated through LOOCV analysis, supporting its potential utility in clinical practice [21,27]. NLR, PLR, SII, SIRI, and MHR are calculated from overlapping hematologic components such as neutrophils, lymphocytes, and platelets, which naturally introduces some correlation; however, the use of LASSO regression minimizes the impact of correlated predictors, supporting the robustness of our predictive models.

As mentioned above, we think that inflammation plays a major role in the etiopathogenesis of the facial nerve. Indeed, when we look back at the studies we mentioned above, hematological inflammatory parameters were found to be lower in patients whose Bell’s palsy had completely recovered compared to those who did not. Similarly, in our study, NLR and PLR levels were found to be lower in cases that responded to treatment compared to cases that did not respond. With these findings, we can say that NLR and PLR levels are important parameters in the diagnosis and prognosis of Bell’s palsy. In addition, we confirmed these findings and our argument with the analysis we conducted with the LOOCV method in order to establish it on a more solid ground.

Composite indices like SII and SIRI integrate multiple inflammatory cell lines, potentially providing a more sensitive reflection of systemic inflammation than individual markers. Recent studies in neuroinflammatory and cardiovascular diseases highlight their superior prognostic value [21,31,32]. Our observation that these indices differ significantly between responders and non-responders supports their application as prognostic biomarkers in Bell’s palsy. In the present study, SII and SIRI levels were found to be higher in the patient group compared to the control group. SII and SIRI were found to be significantly different in patients who responded to treatment compared to patients who did not. Since these indices are formed by dividing the increasing parameters by the decreasing parameters in the case of inflammation, they may be more sensitive compared to hematological inflammatory parameters. The clinical relevance of these accessible and cost-effective markers lies in their potential to aid early diagnosis, risk stratification, and individualized treatment monitoring. Identifying patients with heightened systemic inflammation may allow clinicians to tailor therapeutic approaches and follow up more effectively.

Abnormal lipid levels have been shown to potentially contribute to facial nerve inflammation observed in Bell’s palsy [6]. Koc et al. [6] investigated the association between lipid profile parameters and the severity of Bell’s palsy. They found that higher levels of total cholesterol, LDL-C, and triglycerides were significantly correlated with more severe cases of Bell’s palsy [33]. HDL-C is known to exert anti-inflammatory properties, while elevated monocyte levels reflect an increased inflammatory response. Consequently, the MHR has been proposed as a novel indicator of systemic inflammation. MHR is recognized not only as a marker of inflammation but also as a prognostic factor in various cardiovascular conditions [34,35,36,37,38,39]. Moreover, the MHR has emerged as a novel marker linking lipid metabolism to inflammatory status [19,34,35,36,37,38,39]. Elevated MHR has been associated with adverse outcomes in cardiovascular and inflammatory diseases [34,35], and our study extends this to Bell’s palsy, demonstrating its association with disease severity. This aligns with Serifler et al.’s findings that monocyte-related indices reflect the inflammatory milieu in Bell’s palsy [21]. Although MHR has been evaluated in the context of several medical disorders, its role in predicting the severity of Bell’s palsy has not been clearly defined. Our findings are consistent with those of Serifler et al. [21], who demonstrated that elevated monocyte-based inflammatory markers, particularly the MHR, reflect the current inflammatory status in Bell’s palsy. Similar to their results, our study supports the role of MHR as a potential prognostic biomarker, reinforcing the association between systemic inflammation and disease severity in Bell’s palsy. These parameters are not only accessible and cost-effective but also mirror the degree of systemic inflammation. This study uniquely advances the field by integrating multiple hematological inflammatory markers into predictive models for both diagnosis and treatment response in Bell’s palsy, validated through the robust LOOCV method. While previous research has examined individual biomarkers, our approach confirms the combined predictive value and generalizability of these indices, underscoring their potential utility in clinical decision-making and personalized patient management.

The main limitations of this study include its retrospective design, unequal distribution of patients across disease stages, and incomplete data on recent medication use, which may have influenced inflammatory parameters. Time from symptom onset and laterality of paralysis were not systematically recorded and could not be included as covariates. Finally, the single-center design with participants from one region may limit the generalizability of the findings. Standardized examinations at diagnosis and after treatment help mitigate some of these limitations.

### 4.1. Implications for Practice

(i) Hematological inflammatory markers (NLR, PLR, SII, SIRI, MHR) may serve as accessible, cost-effective biomarkers to aid in the diagnosis and prognosis of Bell’s palsy. (ii) Among these, SIRI and MHR demonstrated strong independent predictive value for disease severity and treatment response. (iii) Incorporation of these markers into clinical practice could facilitate early identification of high-risk patients and enable personalized treatment strategies. (iv) Monitoring changes in these indices over time offers a non-invasive approach to assess therapeutic efficacy. (v) Prospective studies are needed to validate these findings and support their routine use in patient management.

### 4.2. Conclusions

This study demonstrates that systemic inflammation, reflected by hematological markers such as NLR, PLR, SII, SIRI, and MHR, is significantly associated with both the pathogenesis and prognosis of Bell’s palsy. These markers were notably elevated in patients compared to healthy controls and correlated with greater disease severity and poorer treatment response. Among them, SIRI was the most powerful and independent predictor, as confirmed by LASSO regression and LOOCV analysis. These easily accessible and cost-effective markers, especially composite indices like SIRI and MHR, may be valuable in the early diagnosis and prognostic assessment of Bell’s palsy. Notably, MHR, which reflects the balance between proinflammatory monocytes and anti-inflammatory HDL, emerged as a promising indicator of disease severity.

## Figures and Tables

**Table 1:** Demographic and laboratory findings of the study population.

Variables	Control Group	Bell’s Palsy Group	*p*-Value
	*n* = 156	*n* = 156	
**Gender, *n* (%)**			
Female	49 (31.4)	52 (33.3)	0.717
Male	107 (68.6)	104 (66.7)	
**Age, years**	47.1 ± 11.4	46.3 ± 13.2	0.580
**Effect side, *n* (%)**			
Left	-	94 (60.3)	-
Right	-	62 (39.7)	
**Severity of palsy, *n* (%)**			
Grade IV	-	68 (43.6)	-
Grade V	-	78 (50.0)	
Grade VI	-	10 (6.4)	
**Laboratory findings**			
Hemoglobin, g/dL	14.8 ± 1.3	14.5 ± 1.6	0.401
RBC, ×10^6^ µL	4.9 ± 0.7	4.8 ± 0.8	0.484
Hematocrit, %	43.4 ± 3.3	43.1 ± 4.8	0.805
MCV, fL	87.3 ± 3.7	87.3 ± 9.7	0.643
MCH, pg	30.3 ± 0.9	30.1 ± 2.0	0.752
MCHC, g/dL	34.3 ± 0.9	34.2 ± 2.1	0.980
Leukocytes, ×10^3^ µL	5.3 ± 1.0	7.8 ± 2.2	<0.001 *
Lymphocytes, ×10^3^ µL	2.2 ± 0.7	2.0 ± 0.3	0.001
Neutrophils, ×10^3^ µL	2.4 (2.1–3.0)	4.2 (3.6–5.1)	<0.001 *
Monocytes, ×10^3^ µL	0.5 ± 0.1	0.6 ± 0.2	<0.001 *
Platelets, ×10^3^ µL	212.3 ± 24.3	242.2 ± 67.1	<0.001 *
MPV, fL	9.9 ± 0.8	9.4 ± 0.9	<0.001 *
PCT, %	0.2 ± 0.1	0.2 ± 0.1	0.854
PDW, %	11.0 ± 1.5	10.5 ± 1.8	0.009 *
CRP, mg/dL	1.4 (0.9–2.3)	4.0 (2.1–6.2)	<0.001 *
PLR	90.6 (81.4–112.2)	115.7 (91.3–140.6)	0.008 *
NLR	1.2 (0.9–1.7)	2.0 (1.5–2.6)	<0.001 *
SII	233.4 (198.4–392.6)	452.2 (339.3–668.4)	<0.001 *
SIRI	0.5 (0.4–0.7)	1.2 (0.9–1.6)	<0.001 *
MHR	0.009 (0.007–0.012)	0.015 (0.011–0.021)	<0.001 *

Data are mean ± standard deviation or median (IQR), or number (%). * *p* < 0.05 indicates statistical significance. **Abbreviations**: CRP, C-reactive protein; MCH, mean corpuscular hemoglobin; MCHC, mean corpuscular hemoglobin concentration; MCV, mean corpuscular volume; MPV, mean platelet volume; NLR, neutrophil-to-lymphocyte ratio; PCT, plateletcrit; PDW, platelet distribution width; PLR, platelet-to-lymphocyte ratio; RBC, red blood cell count; SII, systemic immune-inflammation index; WBC, white blood cell count; MHR, monocyte-to-high-density lipoprotein cholesterol ratio.

**Table 2:** Diagnostic performance of inflammatory biomarkers in Bell’s palsy.

Biomarker	AUC	95% CI	Sensitivity (%)	Specificity (%)
**SIRI**	0.965	0.925–0.985	83.4	80.1
**CRP**	0.865	0.836–0.885	92.3	91.7
**NLR**	0.771	0.724–0.816	78.9	74.4
**SII**	0.849	0.803–0.904	75.0	72.5
**MHR**	0.741	0.696–0.786	80.2	76.3

**Table 3:** Inflammatory markers related to Bell’s palsy as identified by LASSO analysis.

Characteristics	Baseline		
**% variation explained by latent factors**			
For predictor variables (inflammatory mediators)	0.95		
For outcome variables (AR)	0.70		
N of used latent factors	1		
AUC	0.98 (0.95–1.00)		
N of correctly classified	92.1% (88–96%)		
*p*-value	<0.001		
	**Factor**	**VIP**	±
Top inflammatory markers	SIRI	3.97	+
responsible for the outcome	CRP	2.58	+
	MHR	1.41	+

Age, gender were adjusted in all analyses. *p*-values are for the associations between outcome and latent factors. Abbreviations: see Table 1, VIP, variance importance in projection; ±, positive/negative association.

**Table 4:** Comparison of the LASSO method and Leave-One-Out Cross-Validation (LOOCV) method for predicting Bell’s palsy using a multivariable regression model.

Statistics	LOOCV Method		LASSO Method	
	Bell’s Palsy		Bell’s Palsy	
	Yes	No	Yes	No
**Test**				
** Positive**	131	4	132	4
** Negative**	152	25	152	24
**AUC**	0.97		0.98	
**SE**	0.02		0.02	
**95% CI**	0.94–1.00		0.95–1.00	
**Sensitivity, (95% CI), %**	84.0 (77.4–88.9)		84.6 (78.1–89.4)	
**Specificity, (95% CI), %**	97.4 (93.6–99.0)		97.4 (93.6–99.0)	
**PPV, (95% CI), %**	97.0 (92.6–98.8)		97.2 (92.7–98.9)	
**NPV, (95% CI), %**	85.9 (80.0–90.2)		86.4 (80.5–90.7)	
**Correctly classified, (95% CI) %**	90.7 (87.0–93.5)		92.1% (88–96%)	

Age, gender were adjusted in all analyses. **Abbreviations**: NPV, negative predictive value; PPV, positive predictive value; SE, standard error.

**Table 5:** Comparison of demographic and laboratory findings by the House–Brackmann Scoring system in patients with Bell’s palsy.

Variables	Bell’s Palsy		*p*-Value
	Stage IV	Stage V–VI	
	*n* = 128	*n* = 28	
**Gender, *n* (%)**			
Female	41 (32.0)	11 (39.3)	0.461
Male	87 (68.0)	17 (60.7)	
**Age, years**	46.9 ± 12.6	43.6 ± 15.9	0.244
**Effect side, *n* (%)**			
Left	78 (60.9)	16 (57.1)	0.710
Right	50 (39.1)	12 (42.9)	
**Laboratory findings**			
Hemoglobin, g/dL	13.5 ± 1.6	13.5 ± 1.6	0.957
RBC, ×10^6^ µL	4.8 ± 0.8	4.9 ± 0.8	0.452
Hematocrit, %	41.1 ± 4.7	41.2 ± 5.0	0.920
MCV, fL	83.0 ± 10.0	84.4 ± 8.3	0.516
MCH, pg	28.5 ± 1.9	28.1 ± 2.2	0.261
MCHC, g/dL	32.8 ± 2.2	33.2 ± 1.4	0.329
Leukocytes, ×10^3^ µL	7.8 ± 2.3	7.7 ± 2.0	0.875
Lymphocytes, ×10^3^ µL	2.3 ± 0.6	1.8 ± 0.9	0.010 *
Neutrophils, ×10^3^ µL	4.3 ± 1.2	5.5 ± 1.8	0.002 *
Monocytes, ×10^3^ µL	0.6 ± 0.1	0.7 ± 0.2	0.002 *
Platelets, ×10^3^ µL	237.1 ± 66.7	265.3 ± 65.1	0.044
MPV, fL	9.4 ± 0.9	9.4 ± 1.0	0.957
PCT, %	0.2 ± 0.1	0.2 ± 0.1	0.957
PDW, %	10.5 ± 1.8	10.5 ± 1.9	0.957
CRP, mg/dL	4.0 (2.1–6.4)	6.9 (3.8–9.1)	<0.001 *
PLR	100.0 (78.9–130.4)	154.2 (110.4–236.6)	<0.001 *
NLR	1.9 (1.5–2.3)	3.9 (2.2–5.3)	<0.001 *
SII	413.8 (328.4–570.2)	935.3 (770.5–1290.2)	<0.001 *
SIRI	1.1 (0.8–1.4)	2.7 (1.7–3.4)	<0.001 *
MHR	0.013 (0.010–0.018)	0.019 (0.014–0.025)	0.003 *

Data are mean ± standard deviation or median (IQR), or number (%). * *p* < 0.05 indicates statistical significance. **Abbreviations**: CRP, C-reactive protein; MCH, mean corpuscular hemoglobin; MCHC, mean corpuscular hemoglobin concentration; MCV, mean corpuscular volume; MPV, mean platelet volume; NLR, neutrophil-to-lymphocyte ratio; PCT, plateletcrit; PDW, platelet distribution width; PLR, platelet-to-lymphocyte ratio; RBC, red blood cell count; SII, systemic immune-inflammation index; WBC, white blood cell count; MHR, monocyte/HDL Ratio.

**Table 6:** Diagnostic performance of predicting treatment response (recovered vs. non-recovered) in Bell’s palsy.

Biomarker	AUC	95% CI	Sensitivity (%)	Specificity (%)
**SIRI**	0.867	0.818–0.916	87.0	82.1
**CRP**	0.793	0.732–0.854	81.2	75.3
**MHR**	0.741	0.673–0.809	76.4	70.8

**Table 7:** Demographic and laboratory findings in the recovery and non-recovery groups.

Variables	Recovery Group	Non-Recovery Group	*p*-Value
	*n* = 125	*n* = 31	
**Gender, *n* (%)**			
Female	40 (32.0)	12 (38.7)	0.478
Male	85 (68.0)	19 (61.3)	
**Age, years**	47.1 ± 12.5	43.1 ± 15.7	0.130
**Laboratory findings**			
Hemoglobin, g/dL	13.5 ± 1.6	13.6 ± 1.6	0.637
RBC, ×10^6^ µL	4.8 ± 0.9	4.9 ± 0.6	0.687
Hematocrit, %	41.2 ± 4.7	40.9 ± 5.2	0.761
MCV, fL	82.8 ± 10.6	85.1 ± 4.6	0.246
MCH, pg	28.5 ± 1.9	28.3 ± 2.3	0.580
MCHC, g/dL	32.8 ± 2.2	33.2 ± 1.3	0.292
Leukocytes, ×10^3^ µL	6.4 ± 2.2	7.8 ± 2.3	<0.001 *
Lymphocytes, ×10^3^ µL	2.3 ± 0.6	1.8 ± 0.8	<0.001 *
Neutrophils, ×10^3^ µL	4.3 ± 1.2	5.5 ± 1.7	0.001 *
Monocytes, ×10^3^ µL	0.6 ± 0.2	0.7 ± 0.2	0.023 *
Platelets, ×10^3^ µL	239.4 ± 68.0	253.3 ± 63.3	0.304
MPV, fL	9.4 ± 0.9	9.5 ± 0.7	0.536
PCT, %	0.2 ± 0.1	0.2 ± 0.1	0.440
PDW, %	10.4 ± 1.8	11.0 ± 1.7	0.092
CRP, mg/dL	2.4 (2.1–4.8)	3.9 (2.8–6.8)	0.018 *
PLR	112.5 (88.2–135.1)	121.3 (99.7–148.2)	0.045 *
NLR	1.9 ± 0.6	4.0 ± 1.9	<0.001 *
SII	413.3 (320.7–554.9)	873.1 (681.5–1274.5)	<0.001 *
SIRI	1.1 ± 0.4	2.5 ± 1.0	<0.001 *
MHR	0.014 (0.010–0.019)	0.020 (0.015–0.026)	0.002 *

* Data are mean ± standard deviation or median [IQR], or number (%). *p* < 0.05 indicates statistical significance. **Abbreviations**: CRP, C-reactive protein; NLR, neutrophil-to-lymphocyte ratio; PLR, platelet-to-lymphocyte ratio; SII, systemic immune-inflammation index; SIRI, systemic inflammation response index; MHR, monocyte/HDL Ratio.

**Table 8:** Diagnostic performance of treatment response prediction (recovery vs. non-recovery) indicated that SIRI had the highest prognostic accuracy, followed by SII.

Biomarker	AUC	95% CI	Sensitivity (%)	Specificity (%)
**SIRI**	0.89	0.845–0.935	88.2	83.5
**NLR**	0.81	0.754–0.867	83.4	76.2
**CRP**	0.78	0.721–0.839	79.1	72.8
**SII**	0.82	0.765–0.875	85.0	79.4

**Table 9:** Inflammatory and metabolic markers related to non-recovery in patients with Bell’s palsy as identified by LASSO analysis.

Characteristics	Baseline		
**% variation explained by latent factors**			
For predictor variables (inflammatory mediators)	0.97		
For outcome variables (AR)	0.50		
N of used latent factors	1		
AUC	0.86 (0.77–0.95)		
N of correctly classified	93.8% (0.88–96.5%)		
*p*-value	<0.001		
	**Factor**	**VIP**	**±**
Top markers responsible for outcome	SIRI	1.65	+
	CRP	1.44	+

Age, gender were adjusted in all analyses. *p*-values are for the associations between outcome and latent factors. Abbreviations: see Table 1, VIP, variance importance in projection; ±, positive/negative association.

## Data Availability

The data that support the findings of this study are available on request from the corresponding author. The data are not publicly available due to privacy or ethical restrictions.
